# RETRACTION

**DOI:** 10.1002/clc.23357

**Published:** 2020-03-17

Zi‐Heng Zheng et al., Interleukin‐1 blockade treatment decreasing cardiovascular risk. Clin Cardiol. 2019 Oct;42(10):942‐951 (https://onlinelibrary.wiley.com/doi/full/10.1002/clc.23246). The above article, published online on 15 August 2019 in Wiley Online Library (wileyonlinelibrary.com), has been retracted by agreement between the authors, the journal Editor in Chief Dr. John Camm, and John Wiley & Sons Ltd. The retraction has been agreed due to significant errors in the reported meta‐analyses and other errors which do not support the conclusions drawn.

